# Pets in the Digital Age: Live, Robot, or Virtual?

**DOI:** 10.3389/fvets.2015.00011

**Published:** 2015-05-07

**Authors:** Jean-Loup Rault

**Affiliations:** ^1^Animal Welfare Science Centre, Faculty of Veterinary and Agricultural Sciences, University of Melbourne, Parkville, VIC, Australia

**Keywords:** domestication, human–animal relationship, social, technology, communication

Over half the people in Western societies share their daily life with pets, which makes it the norm rather than the exception. Our shared history with domestic animals goes back tens of thousands years. However, technological advances in the last decades – computer, internet, social media – revolutionized our means of communication, and particularly our social lives. A legitimate but tacit question is whether this technological evolution will also change human–animal relationships, and concurrently, the place of pets in human societies. Pet ownership in its current form is likely unsustainable in a growing, urbanized population. Digital technologies have quickly revolutionized human communication and social relationships, and logically could tackle human–animal relationships as well. The question is whether these new technologies actually represent the future of pet ownership, helping tackle its sustainability while solving animal welfare issues.

To consider whether new technologies could substitute animal use, one should first consider the reasons for keeping animals, and particularly pets. Domestication started some 18,000–32,000 years ago with dogs. However, today’s pets cover a wide range of species from mammals, birds, and fish to the more ‘exotic’ reptiles, arachnids, and even insects. One of the many definitions of a pet is “a domesticated animal kept for pleasure rather than utility” (Merriam-Webster Dictionary), although non-domesticated species are increasingly popular. The benefits or function that humans derive from pet ownership are still debated. It may be a cultural habit: “I had a pet growing up, so it is normal to have one,” even though for some people the only interaction with their pet is restricted to providing food and water and no other forms of social interaction, hence only partly fulfilling our ‘duty of care.’ Pet ownership could be a sign of status: dog ownership can be interpretated as an economic indicator, highly correlated with rise in countries’ income. Pets may be used to compensate for lack of social relationships, as pet owners report feeling less lonely, although there are evidence that pets facilitate human social interactions. A widespread theory holds that pet ownership brings health benefits. Alternative explanations are, for instance, that pet ownership may improve reproductive fitness given that people more easily approach someone walking their dog. Nevertheless, some scientists remain dubious, citing inconsistent findings on the benefits of pet ownership (1). Irrespectively, the human–animal relationship is a strong and emotional powerful bond: pets are often considered part of the family, and dogs and children activate common brain regions in mothers (2), drawing on the hormone oxytocin (3). Historically, the human–animal relationship has already been a changing concept, from animals for their consumptive value to ‘reservoirs of human need’ such as love and care (4). However, we are possibly witnessing the dawn of a new era, the digital revolution with likely effects on pet ownership, similar to the industrial revolution which replaced animal power for petrol and electrical engines.

Everyone is familiar with the digital era. While only the most recent generations were born in it, others have had to adapt to these new technologies. This digital revolution has been a fast change at the scale of human evolution. In a matter of two decades, new means of communication and social media have drastically changed our own social relationships (e.g., Facebook with over 1 billion active users), for better or worse. Since our relation to animals is embedded in humanity (5), there is no reason to expect that it will be spared by this digital phenomenon. In fact, the current technological revolution has quickly resulted in cultural shifts, and operates at a much faster pace than the classic domestication selection process. The question then is what will be the place of pets in a digitally driven human society?

The conundrum of pet ownership can be addressed according to the concept of the 3Rs (6): refinement – not all species are suitable or animal use ethical; reduction – pet ownership as luxury, remote interactions through technology; replacement – robot, virtual reality. Hence, I decided to expand on these three options: animals used in a different way, less animals, or no animals. These avenues have been studied under convergent endeavors in different fields. Alternatively, pet numbers could increase, as seen with the boom in internet trade, although pet ownership as we know it is likely to change due to sustainability and animal welfare concerns.

Refinement could involve restricting animal use allowed by society. As an illustration, keeping highly sentient or intelligent species in captivity is becoming increasingly questioned, as we are unable to fulfill their social or mental needs (e.g., primates, cetaceans) (7). Hence, ownership of some species may become socially unacceptable, such as for singly kept parrots. This view is probably not so reliant on technological advances as ethical change progresses on its own, partly driven by cultural change (4). Nevertheless, technologies could improve animal welfare by providing cognitively demanding tasks or other forms of environmental enrichment, or conversely helping us elucidate animals’ cognitive and mental needs. Technologies could also help facilitate interactions such as remote communication between owners and their dogs left alone at home (8).

Reduction is an interesting proposal as there is an inherent conflict between our remoteness from nature, which appears to stimulate pet keeping (the ‘biophilia’ concept), and sustainability of pet keeping in a growing, urbanized society. Pets are common in Western cultures and on the rise in ‘developing’ countries such as in Asian countries. Yet, it is difficult to imagine how more than half of the 9.6 billion people of 2050 could still keep pets. Efforts to develop cities designed to be green and pet-friendly are ongoing. However, a more realistic future is that pets may become a luxury possession for people who can afford to sustain their cost and fulfill their needs in terms of space, social, and mental needs according to possibly higher ethical standards raised by future societies. Conversely, the fast-changing hypes and trends around particular pet breeds or species, which then become suddenly unpopular, may worsen the issue of ‘unwanted’ pets. Alternatives that have been investigated to date are remote interactions through the internet with farm animals, for example, in an attempt to reconnect people with the origin of their food and transparency (9).

The last option is most intriguing, that technologies could allow us to replace animal use. There are already alternatives to the use of animals in research, with *in vitro* or computer models, with synthetic fibers to replace animal-derived fibers (e.g., wool), and in its infancy for *in vitro* meat production from cells. However, the question of whether technologies could replace pets has been relatively unexplored. The Tamagotchi, a handheld egg with a digital screen that one had to feed and take care of, can probably be considered the precursor of the artificial pet movement in 1996. The rationale behind its rudimentary design was that the physical appearance was not as important as the personal relationship the owner had with it. Nevertheless, the ‘life’ of a Tamagotchi could be considered rather precarious, with repeated deaths upon neglect in disproportionate measures to (fortunately) real live pets. Further research focuses on identifying the features required by social robots to simulate live interactions for which both psychology and ethology are useful approaches (10). The Sony AIBO robotic pet dog is probably the most well-known commercialized attempt (11), but countless other patents have been submitted. Interestingly, children treat the AIBO robotic dog as if it was a living dog, and this does not vary by a child’s attachment to a pet at home or involvement in computer technology (11). Another interesting approach is Paro, a robotic baby seal aimed to elicit positive responses from patients and classified as a medical device in the USA, with a design intentionally based on an unfamiliar animal to overcome the expectations people may have from the behavior of more familiar animal species. This illustrates another advantage of robotic pets as substitutes in situations where live pets are undesirable (e.g., old or allergic people). Overall, robotic pets appear to elicit similar responses from humans as live pets (11), but it is unclear whether they stimulate identical responses and replace that need for a pet; notwithstanding that, scientists are still debating the function and benefits humans derive from (live) pets.

Progress is ongoing to computer-simulate interactions with a pet through virtual reality (12), similarly to the attempt to computer-simulate human social interactions. Virtual reality can be defined as a computer-generated artificial environment that is affected by the actions of a person who is experiencing it. Hence, it potentially can fulfill all aspects of a human–animal interaction apart from physical contact. Virtual worlds involving animals are, for instance, HappyFarm, a social network online game based on farm animal management simulation, which regrouped 23 million daily users at the height of its popularity, aquarium virtual fish tanks, and numerous adaptations of these principles. Similarly to the robotic quest, central questions remain to elucidate the features that make for an appealing pet (marketing interest), and the function that this virtual pet fullfills (scientific interest). While Nintendogs, a screen-based virtual pet console game, offers some companionship, it was evaluated as significantly less than the companionship derived from a real dog or cat (13). Another study showed that children associated a stuffed dog with friendship, but a virtual dog with entertainment (14).

On the one hand, several aspects still differ between today’s virtual and robotic pets compare to live pets. The responsibility that we feel for each may differ, as suggests the difference between the lifetime of a Tamagotchi and that of a live pet. Whether this responsibility through our duty of care is linked to the satisfaction of pet ownership is unknown. However, we commonly hear that kids learn a greater sense of responsibility growing up around pets, and this is used in school programs. It also remains challenging to simulate the natural unpredictability of interacting with a live animal. Nevertheless, people seem to attribute to more advanced robots, like the Sony AIBO dog a status of their own, between that of animals and artifacts (15).

On the other hand, movies such as “Her”’ in which a man falls in love with a computer-generated female operating system show the potential dangers of virtual reality to the human brain with its evolutionary attachment process. Patients using the Paro robot reported that they “know it is not real but still love it,” and talk to it as a living being. Hence, robots can without doubt trigger human emotions. Indeed, funerals are held for AIBO robotic dogs in Japan nowadays, since Sony recently closed their last tech ‘clinic’ used to fix them. If artificial pets can replicate the human benefits obtained from live pets, does that mean that the human–animal emotional bond is solely dependent on ourselves and the image that we project on a live or artificial interactive partner? Does it ethically matter if the benefits of keeping artificial pets outweight the risks, sparing other live pets’ potential animal welfare issues? Conversely, could artificial pets make future human generations insensitive to the treatment of live animals (15)? As Turkle (16) mentioned, “we make our technologies, and they, in turn, shape us.” Others are warning about these fast technological advances, with Stephen Hawking fearing that humans, limited by slow biological evolution, could not compete and would be superseded by full artifical intelligence, and Bill Gates warning that artificial intelligence poses a real threat to humankind. What risks would artificial pets cause to humans compared to live pets? And to domestic animals?

The pace of artificial pet development, and underlying research, remains in its infancy with much to be discovered. At present, artificial pets can be described as mediocre substitutes for live counterparts. Yet, quick technological progress is to be expected, and this phenomenon raises many ethical questions. Are animals what make us humans? Or are we witnessing a leap into what domestication always was: to select animals to be perfect pets, with a need to update the definition of pets as an animal or an artificial device kept for pleasure? Animal welfare science, a field that emerged in the 1960s, will likely have to follow this radical change in our relation to animals. This may be for the best with a more egalitarian and considerate attitude for life. However, are ethologists and veterinary scientists missing on the largest revolution in human–animal relationship history, its development, and consequences? “Let the future tell the truth,” to quote the futuristic scientist Nikola Tesla (17).

## Conflict of Interest Statement

The author declares that the research was conducted in the absence of any commercial or financial relationships that could be construed as a potential conflict of interest.
